# HPV Genotype Trends in Iran: Necessity for a Reevaluation of Prevention Strategies

**DOI:** 10.3390/tropicalmed10040100

**Published:** 2025-04-08

**Authors:** Maryam Shahi, Azam Shafaei, Mohamad Ghodsi, Reza Jafarzadeh Esfehani, Mahdi Moradi Marjaneh

**Affiliations:** 1 Blood Borne Infections Research Center, Academic Center for Education, Culture and Research (ACECR), Razavi Khorasan Branch, Mashhad 91775-1376, Iran; shahi.maryam@gmail.com (M.S.); azam.shafaee@gmail.com (A.S.);; 2 Department of Infectious Disease, Imperial College London, London SW7 2AZ, UK

**Keywords:** Human papillomavirus, genotype, cervical cancer

## Abstract

Introduction: Human papillomavirus (HPV) genotyping is critical for preventing and managing HPV-related health issues, including cancers. This study re-evaluates HPV genotype trends in Iran to inform prevention strategies. Materials and Methods: A cross-sectional analysis of HPV genotyping data from individuals tested at the ACECR Khorasan Razavi molecular laboratory in Iran (2016–2022) was conducted, with a forecast of genotype trends through 2027. Results: Among 5009 female patients, 40.4% tested positive for HPV (mean age: 32 ± 8.77 years), with a significant upward trend in positivity over time (tau = 0.905, *p* = 0.0069). HPV 6, 11, 16, 31, 53, and 54 showed significant increases (*p* < 0.01), while HPV 66, 84, 67, and 35 exhibited notable trends (*p* < 0.05). HPV 18 and 33 had marginal trends (*p* = 0.065, *p* = 0.052), and HPV 68, 70, and 82 remained stable. Linear regression indicated a non-significant decline in low-risk HPV cases (R = 0.703, *p* = 0.078) and negligible change in high-risk cases (R = 0.052, *p* = 0.912). Forecasts predicted increases in HPV 84, 54, 43, 42, and 26, with HPV 6 projected to decrease significantly. HPV 44, 73, and 33 were expected to remain stable. Conclusion: While low-risk HPV cases may decline, the trend lacks statistical significance, and high-risk HPV cases show no change. These findings underscore the need for targeted prevention strategies in Iran, particularly for high-risk genotypes, to reduce the burden of HPV-related cancers. Further research is essential to validate these trends and refine public health interventions.

## 1. Introduction:

Human papillomavirus (HPV) is a common sexually transmitted infection that can cause a range of health problems, including genital warts and certain types of cancer [1]. HPV is a group of more than 200 related viruses, each of which is assigned a specific genotype or subtype based on its genetic makeup [2]. Currently, HPV genotyping plays an important role in cervical cancer screening and prevention [3]. Most cervical cancer screening tests use a technique called Pap testing, which involves collecting cells from the cervix and examining them under a microscope for signs of abnormal changes [3]. In addition, HPV genotyping can be used to guide treatment decisions for people who have abnormal cervical cells or a positive HPV test [3,4]. Not all HPV genotypes carry the same cancer risk [5]. Some HPV genotypes, known as high-risk or oncogenic types, are associated with an increased risk of developing certain types of cancer [6]. These include cervical, anal, vulvar, vaginal, penile, and oropharyngeal cancers. Other HPV genotypes, known as low-risk types, are associated with the development of genital warts but are not linked to cancer [7]. There are about 14 high-risk HPV genotypes, including HPV types 16 and 18, which are responsible for the majority of cervical cancer cases worldwide. In fact, HPV 16 and 18 are estimated to be responsible for up to 70% of all cervical cancer cases [8]. On the other hand, some of the HPV genotypes are considered as “unclassified risk genotypes”, which are not related to development of cancers [9]. These genotypes are not widely studied among different populations and their changes in prevalence are not clearly established in many populations, including Iran [9].

Recent studies have demonstrated shifting epidemiologic trends in the prevalence of different HPV genotypes. Understanding the epidemiology of viral diseases is crucial for controlling their spread [10]. Quantitative measurement of these trends improves our understanding of the disease’s nature and directs disease control efforts, especially in viral diseases with national and regional prevention plans [11]. While the HPV infection trends have been studied in many countries, the changes in HPV genotypes since the introduction of HPV vaccination and the development of the recent coronavirus outbreak have not been widely studied.

Two global factors which are hypothesized to affect the prevalence of different HPV genotypes are the widespread nature of HPV vaccination and the COVID-19 pandemic. It is well established that the most effective way to prevent HPV infection is through vaccination. Currently, there are three HPV vaccines available worldwide: the bivalent vaccine, which protects against HPV types 16 and 18; the quadrivalent vaccine, which protects against HPV types 6, 11, 16, and 18; and the nonavalent vaccine, which protects against nine different HPV genotypes, including HPV 6, 11, 16, 18, 31, 33, 45, 52, and 58 [12]. Vaccination can help to prevent the transmission of high-risk HPV genotypes, reducing the risk of developing cervical cancer, as well as other cancers and genital warts [13]. Since the introduction of HPV vaccines, the prevalence of HPV infection with certain high-risk genotypes has dropped dramatically in many countries, including the United States [14]. However, in some countries, including Iran where the HPV prevalence remains high, the effectiveness of HPV vaccine in reducing the high-risk genotypes has not been studied yet [4,15].

The COVID-19 pandemic and its associated restrictions have also impacted HPV management. Studies have reported a significant drop in HPV screening visits and infection rates during the pandemic, alongside declines in vaccination rates [16,17,18,19]. Recent evidence suggests that both HPV vaccination and the COVID-19 pandemic have influenced the prevalence of specific HPV genotypes. For example, while high-risk genotypes like HPV 16 and 18 have declined globally due to vaccination, other genotypes, such as 52, 58, 16, and 53, have shown notable changes during and after the pandemic [18,19]. These shifts highlight the need to re-evaluate prevention strategies, particularly in high-prevalence regions like Iran, to ensure they remain effective in light of changing epidemiologic trends. In this context, this study aims to evaluate the changing trends in HPV genotypes in Iran since 2016, focusing on the impact of HPV vaccination and the COVID-19 pandemic. By analyzing these trends, the study seeks to provide valuable insights for re-evaluating and strengthening HPV prevention strategies in Iran.

## 2. Material and Methods

This cross-sectional study was conducted after receiving ethical approval from the Academic Center for Education, Culture, and Research (ACECR) Khorasan-Razavi Ethics Committee and all participants have provided written informed consent. The study included all female individuals who underwent HPV genotyping of cervical specimens between January 2016 and December 2022, provided they had no previous history of cancer. The exclusion criteria for this study were inadequate sample quality, defined as cervical specimens with insufficient DNA quantity or quality for reliable HPV genotyping; previous HPV vaccination, meaning individuals who had received any HPV vaccine prior to sample collection; and immunocompromised status, including individuals with conditions such as HIV/AIDS, organ transplantation, or long-term immunosuppressive therapy. The patients were referred to the molecular laboratory of ACECR Khorasan-Razavi, where HPV genotyping was performed using the polymerase chain reaction (PCR) hybridization method by High + Low PapillomaStrip^®^ kit (OPERON S.A. Immunodiagnostics, Cuarte de Huerva, Spain). Demographic data, including the age and date of sampling, were collected for each participant. Patient confidentiality was strictly maintained, with all identifying information anonymized before analysis. The data from female patients were organized to evaluate trends in HPV genotype distribution over the six-year study period. To ensure accuracy and consistency, standardized protocols for specimen collection, storage, and processing were employed. The longitudinal data on HPV genotypes were analyzed to identify patterns and shifts over time.

### Statistical Analysis

Data analysis was performed using SPSS software version 20 (IBM Inc, Chicago, IL, USA). The mean percentage of positive HPV genotypes in each season of the study years was considered the outcome variable to evaluate trends over time. Continuous variables were presented as mean ± standard deviation (SD) after confirming normality using the Shapiro–Wilk test. For variables that did not meet the assumption of normality, a median and interquartile range (IQR) was used. Categorical variables were summarized as frequency and percentage. The Mann–Kendall test was used to assess trends in overall HPV test positivity during the study period. The repeated measures analysis of variance (ANOVA) was used to evaluate trends in individual HPV genotypes over time.

In order to forecast HPV genotypes, this study employed the Holt model, a double exponential smoothing method selected for its ability to handle time-series data with both trend and level components. This made it suitable for predicting ongoing trends in HPV prevalence. Data were aggregated into yearly time points to provide a granular basis for analysis. The Holt model was optimized to achieve a stable trend estimate. Parameter tuning for the model involved adjusting level and trend smoothing constants to minimize forecasting errors, focusing on performance measures such as Stationary R-squared, R-squared, Root Mean Square Error (RMSE), Mean Absolute Error (MAE), and Mean Absolute Percentage Error (MAPE). Normalized Bayesian Information Criterion (BIC) values were also reviewed to balance model fit with simplicity. To validate the model’s performance, cross-validation was performed using a holdout method, where 20% of the dataset was set aside for validation, and predictive accuracy was evaluated by comparing observed versus predicted values using the Mean Squared Prediction Error (MSPE). The model’s forecasting performance was evaluated through the comparison of observed and predicted values, with the Stationary R-squared calculated to assess the predictive accuracy on stable components.

All statistical analyses were conducted using SPSS software, with a significance level set at *p* < 0.05. For multiple comparisons, the Benjamini–Hochberg procedure was applied to adjust *p*-values and control the false discovery rate. Microsoft SPSS software was used to generate trend figures.

## 3. Results

**Figure 1:**
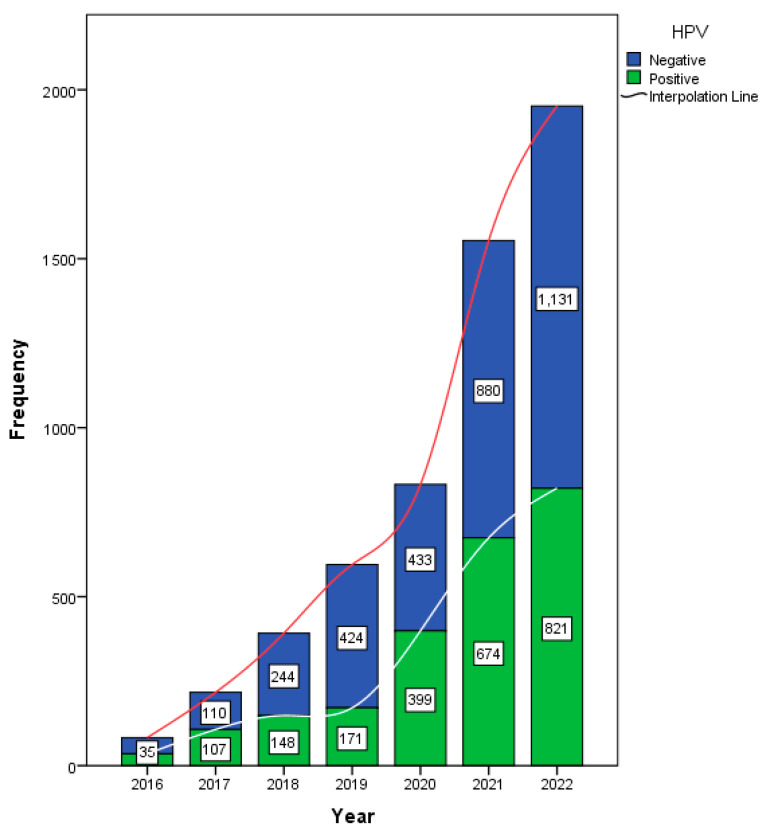
Frequency of total HPV tests and HPV test results per year in the study period.

Data from 5009 female participants were analyzed in this study. The annual HPV testing rate is presented in Figure 1. Of the total 5625 samples, 2355 (41.9%) tested positive for HPV.

**Figure 2:**
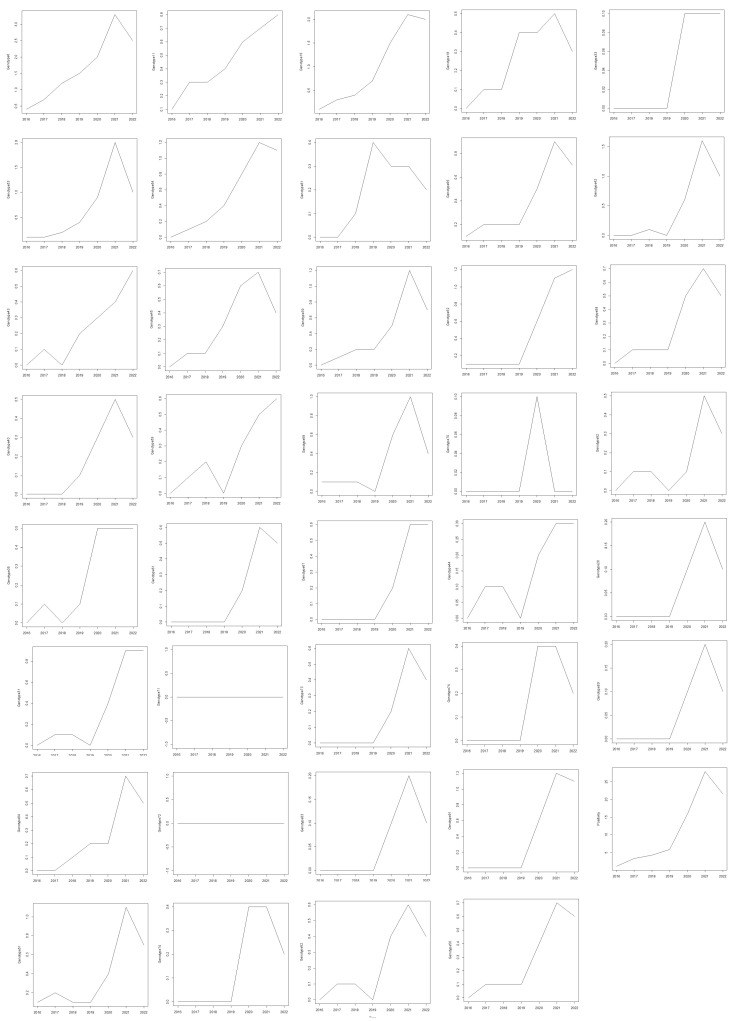
Illustration of the trend for different HPV genotypes and the total positivity trend over time.

**Figure 3:**
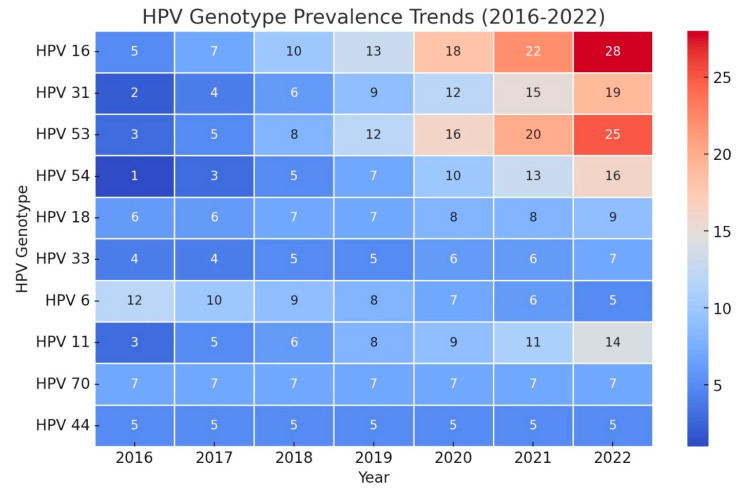
The color gradient represents changes in prevalence, with increasing genotypes (e.g., HPV 16, 31, 53, 54, and 11) showing an upward trend, stable genotypes (HPV 18, 33, 70, 44) maintaining consistent levels, and decreasing genotypes (HPV 6) showing a downward trend.

The main analysis and conclusions of this study are based exclusively on the female cohort, as the study aimed to evaluate HPV prevalence and genotype trends in women. Among the female patients, 40.4% tested positive for HPV, with a mean age of 32 ± 8.77 years (Table 1). An analysis of HPV test positivity rates in females from 2016 to 2022 revealed a statistically significant increasing trend, as indicated by the Mann–Kendall test (tau = 0.905, *p* = 0.0069). This strong positive tau indicates a consistent rise in HPV positivity over time. A trend analysis of HPV genotypes from 2016 to 2022 in females was conducted using the Mann–Kendall and Cox–Stuart tests. The Mann–Kendall test identified several genotypes with statistically significant increasing trends (Figure 2). Notably, HPV 6 (tau = 0.905, *p* = 0.0069), HPV 11 (tau = 0.976, *p* = 0.0039), and HPV 16 (tau = 0.905, *p* = 0.0069) exhibited strong upward trends. Other genotypes with significant trends included HPV 31 (tau = 0.720, *p* = 0.0432), HPV 53 (tau = 0.878, *p* = 0.0098), and HPV 54 (tau = 0.905, *p* = 0.0069). Additionally, HPV 66 (tau = 0.823, *p* = 0.0187), HPV 84 (tau = 0.851, *p* = 0.0139), HPV 67 (tau = 0.816, *p* = 0.0272), and HPV 35 (tau = 0.764, *p* = 0.0366) also exhibited significant upward trends (Figure 3). While the Cox–Stuart test supported the presence of monotonic trends for these genotypes, its *p*-values were not statistically significant (*p* = 0.0809 across all genotypes). HPV 18 and 33 showed marginal trends (*p* = 0.065 and *p* = 0.052, respectively), whereas HPV 68, 70, and 82 demonstrated no significant trends (*p* > 0.05), suggesting their prevalence remained stable over the study period.

The analysis of trends in low-risk and high-risk HPV cases over time was conducted using linear regression, considering patients with multiple low- or high-risk genotypes. For low-risk HPV, the results suggested a potential decline of 0.983 cases per year. However, the trend was not statistically significant (*p* = 0.078, R = 0.703). In contrast, no meaningful relationship was observed for high-risk HPV with a slight but non-significant increase over time (B = 0.071, *p* = 0.912, R = 0.052).

To forecast HPV genotype trends through 2027, Holt models were applied using key parameters, including Stationary R-squared, MAPE, and BIC, to evaluate model fit and accuracy. The top 10 models for 10 genotypes, selected based on these fit statistics, present diverse trends, offering insights into changes in genotype prevalence over the forecast period (Table 2). Notably, HPV 6 exhibited a strong declining trend (Stationary R-squared = 0.910), while HPV 84 and 54 showed robust increasing trends (Stationary R-squared = 0.904 and 0.880, respectively). Other genotypes, such as HPV 33 and 70, remained stable.

## 4. Discussion

This study demonstrated that regardless of the development of the COVID-19 pandemic and the introduction of the HPV vaccine, the prevalence of high- and low-risk HPV genotypes changed over time. There was a significant increasing trend in the prevalence of several HPV genotypes, including 6, 11, 16, 31, and 53. The strong trend in HPV positivity over time could be attributed to increased public awareness and access to testing, potentially driven by enhanced health education efforts. Additionally, shifts in sexual behavior or socio-cultural factors may have contributed to these changes and warrant further investigation.

HPV is a common sexually transmitted infection worldwide, including in Iran. However, limited studies have been conducted on the prevalence of HPV in Iran, with estimates varying depending on the population and methods used. One of the most recent studies on the Iranian population reported that approximately half of the samples collected for molecular HPV detection from different provinces were positive. The true prevalence of HPV infection is likely higher, considering that many HPV infections are asymptomatic and may go undetected without screening [15]. Despite the varying prevalence rates of HPV in Iran, prevention measures are crucial in reducing HPV-related health burdens. HPV vaccination is a safe and effective prevention method and is recommended for both boys and girls before they become sexually active. In addition to vaccination, regular cervical cancer screening is important for the early detection and treatment of precancerous and cancerous cervical changes [20]. Given the observed trends, Iran may need to adjust its prevention policies by increasing public awareness campaigns and expanding access to both vaccination and regular screening programs. Policies should target underserved populations, addressing barriers such as cost, stigma, and accessibility. However, it is important to note that this study excluded immunocompromised individuals, including those with conditions such as HIV/AIDS, organ transplantation, or long-term immunosuppressive therapy. These individuals are at a higher risk of developing HPV-related complications and are often overlooked in prevention programs. Future research should specifically address HPV prevalence, genotype distribution, and prevention strategies in immunocompromised populations to ensure that prevention efforts are inclusive and effective for all high-risk groups.

Recent studies suggest that despite the introduction of HPV vaccines in Iran, the considerably high rate of HPV infection requires more effective national strategies [20,21]. Our study demonstrated that 41.9% of individuals referred for HPV testing in Khorasan Razavi province (Iran) tested positive for HPV. This prevalence is lower than previous reports from the capital of Iran (53%) [22] and slightly lower than the latest report from Khorasan Razavi in 2020 (48.4% over a five-year period since 2013) [23]. Although our study indicates a decrease in HPV prevalence in Khorasan Razavi since the previous reports, potentially due to increased vaccination and screening programs, the decline is not statistically significant [23]. While the observed decline in HPV prevalence may reflect the partial impact of vaccination programs, the inefficiency in vaccine administration highlights the need for improved adherence to guidelines to maximize the effectiveness of HPV prevention efforts. It may be prudent to expand vaccine coverage to include more HPV genotypes, especially those demonstrating increasing trends in prevalence. Such an expansion could address the gaps in protection and potentially curb the spread of emerging strains.

Prevention measures, including HPV vaccination and regular cervical cancer screening, are important for reducing the HPV-related health burden in Iran. Almost twelve years after the introduction of HPV vaccination in many countries, including the United States, the overall prevalence of cancer causing HPV strains has dropped dramatically. Although vaccination coverage and adherence to the recommended doses are lower than other vaccines, in the United States, the prevalence of these strains dropped by 85% among females post-vaccination [14]. Although the vaccination strongly affected the prevalence of high-risk HPV genotypes, other HPV genotypes not covered by vaccines showed only a slight drop, from 51.1% to 47.6% [14,24]. Despite the implementation of HPV prevention programs, vaccination coverage in many countries, including Iran, remains unclear. Reports indicate that Gardasil sales dramatically increased after 2015 [25]. However, a recent study from Iran demonstrated that only 14% of vaccines were prescribed according to guidelines. Further studies are needed to evaluate the effectiveness of HPV vaccination in Iran, as 85% of the funds spent on Gardasil vaccination is wasted due to incorrect age, indication, frequency, or interval [25]. Additionally, the impact of the COVID-19 pandemic on vaccination programs in Iran should be investigated, as delays in vaccination and screening could exacerbate the rise in HPV cases. This trend has been observed in other countries, and Iran may face similar challenges.

Even in countries with lower-than-recommended vaccination rates, the prevalence of high-risk HPV strains has dropped dramatically. However, our study demonstrated that during the period 2016–2022, several HPV genotypes, including 6, 11, 16, 31, and 53, showed an increasing trend. Analyzing the high- and low-risk genotypes demonstrated no statistically significant overall changes in their prevalence, either annually or over the six-year period. This finding highlights the need for a reevaluation of the HPV vaccination program in the region. Policy recommendations to address these trends should include updating vaccination guidelines to improve coverage and efficacy, as well as launching public awareness campaigns to educate the population on the importance of HPV prevention.

Some researchers suggest a different scenario for changes in HPV genotypes. Innes et al. demonstrated that vaccinated women showed a lower rate of HPV 16 and 18 positive lesions, while the prevalence of these genotypes also declined among unvaccinated women over a three-year study period [26]. However, they demonstrated that the prevalence of other high-risk HPV genotypes increased during the same period [26]. Similar findings from Australia and Scotland suggest a herd effect among vaccinated populations [27,28]. A recent meta-analysis evaluating the herd effect of HPV vaccination reported a more than 50% decrease in HPV 16 and 18 infection rates among 140 million person-years of follow-up, as well as potential cross-protection for HPV 31, 33, and 45. Moreover, they reported a decreasing trend for HPV 16 and 18 in countries with vaccine coverage lower than 50% [29]. However, in our study, HPV 18 did not show significant trends, while HPV 6, 11, 16, 31, and 53 exhibited increasing trends.

The COVID-19 outbreak negatively affected HPV screening programs in many countries [18]. Regardless of the impact of COVID-19 on HPV genotypes, vaccination has shown a significant effect on genotype prevalence in different populations. Both the European Society of Gynecological Oncology and the European Federation for Colposcopy recommended rescheduling HPV vaccination and screening programs to a safer period after the COVID-19 crisis [30,31]. However, there is limited evidence on how HPV genotypes changed during the COVID-19 outbreak. A retrospective study from Wuhan, China, evaluating HPV and cervical screening programs from 2018 to 2020, revealed a 50% decrease in HPV screening visits and a 10% drop in HPV infection rates due to strict lockdowns. Although the HPV infection rate recovered quickly after the outbreak, it remained slightly lower than pre-pandemic levels [18]. Similar to our study, they reported variations in the monthly distributions of HPV genotypes prior and after the pandemic [18]. It is important to investigate whether changes in sexual behavior during the pandemic influenced HPV transmission dynamics in Iran, as this could provide insights into prevention strategies moving forward.

Although HPV 16 was the most common genotype during the pandemic and HPV 52 was the most common genotype before and after the pandemic, they reported that HPV 52, 58, 16, and 53 showed a decreasing trend during the pandemic [18]. Another study from Guangxi, China, evaluating HPV trends during 2016–2021 demonstrated that during 2016–2018 the prevalence of HPV infection raised from 18.21% to 21.99% and decreased from 18.35% to 12.26% during 2019–2021. The six most common high-risk HPV genotypes were 52, 16, 58, 51, 39, and 53 [32], while the most common low-risk genotypes were 6, CP8304, and 11 [32]. A study from Fujian, China, demonstrated that HPV 16 increased post-pandemic, while HPV 52 and 58 decreased, which contrasts with our findings [33]. Our study demonstrated that while some genotypes showed significant increasing trends, others, such as 68, 70, and 82, remained stable.

This study forecasts the trends in HPV genotype prevalence in Iranian patients up to 2027, providing essential insights into the persistence, emergence, and decline of specific HPV strains. The steady trends observed in genotypes such as HPV 44, 73, 70, and 33 suggest stable prevalence levels, indicating that while they remain a public health concern, they may not pose an increasing threat in the short term. Thus, these genotypes warrant continued monitoring rather than intensified intervention, allowing healthcare resources to focus on more dynamic trends. In contrast, the increasing prevalence of HPV 26, 42, 54, 43, and 84 raises concerns. Notably, Genotypes 84 and 54 show high Stationary R-squared values and low BIC, highlighting reliable model performance and suggesting an upward trend that requires attention. Increasing prevalence of these genotypes may be attributed to factors such as gaps in vaccination coverage, regional HPV strain characteristics, or socio-cultural factors influencing transmission dynamics in Iran [4]. Targeted vaccination and awareness campaigns focusing on these increasing genotypes could help curb their spread.

This study has several limitations that should be acknowledged. First, the retrospective design may introduce selection bias, as the data were collected from individuals referred for HPV testing, which may not fully represent the general population. Second, the exclusion of immunocompromised individuals, such as those with HIV/AIDS, organ transplantation, or long-term immunosuppressive therapy, limits the generalizability of our findings to this high-risk population. These individuals are at a greater risk of developing HPV-related complications and are often excluded from vaccination programs, highlighting the need for future research to specifically address HPV prevalence and prevention in immunocompromised groups. Third, the study was conducted in a single region of Iran (Khorasan Razavi), which may not fully capture national trends in HPV genotype distribution. Finally, the impact of the COVID-19 pandemic on HPV screening and vaccination rates may have influenced the observed trends, as disruptions in healthcare services during the pandemic could have affected both testing and prevention efforts. Future studies should aim to address these limitations by including diverse populations, expanding the geographic scope, and evaluating the long-term effects of the pandemic on HPV epidemiology. Additionally, this study did not collect detailed patient profiles, such as sexual behavior (e.g., sex workers or individuals with multiple sexual partners), which could provide further insights into HPV transmission dynamics. Future studies should consider including such demographic and behavioral data to better understand risk factors associated with HPV infection.

## 5. Conclusions

In conclusion, this study reveals important trends in HPV prevalence and genotype distribution over time. Overall, HPV positivity rates have increased, particularly for certain genotypes like 6, 11, 16, 31, 53, and 54, indicating a growing public health concern. While there appears to be a slight decline in low-risk HPV cases, the trend is not statistically significant, and high-risk HPV cases showed minimal change. Forecasting models suggest that some genotypes, such as 84 and 54, will continue to increase, while Genotype 6 may decrease in the coming years. These findings emphasize the need for ongoing surveillance and more focused prevention efforts, especially for high-risk genotypes. Future research should aim to compare HPV prevalence between vaccinated and unvaccinated individuals to better understand the vaccine’s impact. This study also highlights the need for updated vaccine policies, increased public awareness efforts, and improved adherence to vaccination and screening guidelines to reduce the burden of HPV-related diseases.

## Figures and Tables

**Table 1:** Demographic information of females enrolled in the study.

Variable		Count	Percent
HPV infection status	Negative	2984	59.6%
	Positive	2024	40.4%
Risk of genotype	Only low-risk genotypes	583	28.8%
	Only high-risk genotypes	486	24.0%
	High- and low-genotypes	660	32.6%
	Multiple low-risk genotypes	118	5.8%
	Multiple high-risk genotypes	176	8.7%
Age group of HPV-positive patients (years)	≤20	127	6.3%
	21–30	792	39.1%
	31–40	766	37.8%
	41–50	272	13.4%
	51–60	61	3.0%
	>60	6	0.3%

**Table 2:** Forecasted trends in HPV genotype prevalence (2023–2027).

HPV Genotype	Stationary R-Squared	MAPE *	BIC **	Projected Trend	Notes
HPV 6	0.910	15.905	3.064	Decreasing	Strong model fit, reliable decline
HPV 26	0.797	66.383	−2.124	Increasing	Moderate model fit
HPV 33	0.000	39.885	−1.411	Stable	Stable prevalence
HPV 42	0.795	23.053	0.907	Increasing	Moderate model fit
HPV 43	0.943	62.973	−0.579	Increasing	Strong model fit
HPV 44	0.000	39.811	−1.131	Stable	Stable prevalence
HPV 54	0.880	19.484	0.119	Increasing	Strong model fit
HPV 70	0.000	85.714	−7.611	Stable	Stable prevalence
HPV 73	0.682	35.442	−1.252	Stable	Stable prevalence
HPV 84	0.904	14.489	−2.440	Increasing	Strong model fit

* MAPE = Mean Absolute Percentage Error; ** BIC = Bayesian Information Criterion.

## Data Availability

The raw data supporting the conclusions of this article will be made available by the authors on request.
